# Supplementary material for: Identification and functional analysis of non-coding regulatory small RNA FenSr3 in Bacillus amyloliquefaciens LPB-18
Source: PeerJ. 2023 May 15;11:e15236. doi: 10.7717/peerj.15236 (PMC10194069; doi:10.7717/peerj.15236)
Supplement: Supplemental Information 4 [file peerj-11-15236-s004.zip › KO/CK-vs-T1_map/map05330.html]

KEGG PATHWAY: Allograft rejection - Reference pathway


|  |  |
| --- | --- |
| **Allograft rejection - Reference pathway** |  |

[
Pathway menu
| Organism menu
| Pathway entry
| Show description
| User data mapping
]

Allograft rejection is the consequence of the recipient's alloimmune response to nonself antigens expressed by donor tissues. After transplantation of organ allografts, there are two pathways of antigen presentation. In the direct pathway, recipient T cells react to intact allogeneic MHC molecules expressed on the surface of donor cells. This pathway would activate host CD4 or CD8 T cells. In contrast, donor MHC molecules (and all other proteins) shed from the graft can be taken up by host APCs and presented to recipient T cells in the context of self-MHC molecules - the indirect pathway. Such presentation activates predominantly CD4 T cells. A direct cytotoxic T-cell attack on graft cells can be made only by T cells that recognize the graft MHC molecules directly. Nontheless, T cells with indirect allospecificity can contribute to graft rejection by activating macrophages, which cause tissue injury and fibrosis, and are also likely to be important in the development of an alloantibody response to graft.

|  |  |  |
| --- | --- | --- |
| Reference pathway | 184% 150% 122% 100% 82% 67% 55% | 图片下载 |
